# Unsupervised CT Lung Image Segmentation of a Mycobacterium Tuberculosis Infection Model

**DOI:** 10.1038/s41598-018-28100-x

**Published:** 2018-06-28

**Authors:** Pedro M. Gordaliza, Arrate Muñoz-Barrutia, Mónica Abella, Manuel Desco, Sally Sharpe, Juan José Vaquero

**Affiliations:** 1 Universidad Carlos III de Madrid, Departamento de Bioingeniería e Ingeniería Aeroespacial, Leganés, ES28911 Spain; 2 Instituto de Investigación Sanitaria Gregorio Marañón, Madrid, ES28007 Spain; 3 Centro de Investigaciones Cardiovasculares Carlos III (CNIC), Madrid, Spain; 4 Centro de Investigación Biomédica en Red de Salud Mental (CIBERSAM), Madrid, ES28029 Spain; 5 Public Health England, Microbiology Services Division, Porton Down, SP4 0JG England

## Abstract

Tuberculosis (TB) is an infectious disease caused by Mycobacterium tuberculosis that produces pulmonary damage. Radiological imaging is the preferred technique for the assessment of TB longitudinal course. Computer-assisted identification of biomarkers eases the work of the radiologist by providing a quantitative assessment of disease. Lung segmentation is the step before biomarker extraction. In this study, we present an automatic procedure that enables robust segmentation of damaged lungs that have lesions attached to the parenchyma and are affected by respiratory movement artifacts in a Mycobacterium Tuberculosis infection model. Its main steps are the extraction of the healthy lung tissue and the airway tree followed by elimination of the fuzzy boundaries. Its performance was compared with respect to a segmentation obtained using: (1) a semi-automatic tool and (2) an approach based on fuzzy connectedness. A consensus segmentation resulting from the majority voting of three experts’ annotations was considered our ground truth. The proposed approach improves the overlap indicators (Dice similarity coefficient, 94% ± 4%) and the surface similarity coefficients (Hausdorff distance, 8.64 *mm* ± 7.36 *mm*) in the majority of the most difficult-to-segment slices. Results indicate that the refined lung segmentations generated could facilitate the extraction of meaningful quantitative data on disease burden.

## Introduction

According to the World Health Organization (WHO)^1^, in 2016, there were 10.1 million incident cases and 1.4 million deaths caused by tuberculosis (TB). More strikingly, latent TB is present in one third of the world’s population. Within this infected population, *Mycobacterium tuberculosis* (Mtb), the causative agent of TB, becomes active in 10% of the cases and mainly damages the lungs owing to its airborne nature. Identification and treatment of latent TB infection could substantially reduce the risk of developing active disease and could be essential if the objective of eradicating TB by 2050 is to be achieved^1–3^.

The classic view of TB as latent or active is inadequate. Recent literature shows that TB manifests as a continuous spectrum between both states^2,4^. Conventional tests used to identify latent TB, the tuberculin skin test and interferon gamma release assay, are indirect markers of exposure to Mtb and indicate a cellular immune response but cannot distinguish between latent and active TB, differentiate reactivation from reinfection, or resolve the various stages within the spectrum of Mtb infection^3,4^.Therefore, better TB biomarkers are needed^5^.

Non-human primates (NHP) have been proven to be clinically relevant models of human disease because of the high level of gene homology which underlies anatomical, physiological and immunological similarities^6–8^. They lead to the development of comparable disease pathology, clinical signs and immune features following Mtb infection. Animal models are fundamental for the development of novel treatments, as they provide a platform in which the efficacy of new interventions can be evaluated against infectious challenge. Longitudinal images of the TB macaque model can be acquired from live animals using medical imaging systems^9–11^ – e.g., chest radiographs (CXR), computed tomography (CT) and position emission tomography(PET) – and employed to visualize the evolution of pulmonary disease.

TB has specific radiological manifestations in chest CT scans that could be used as imaging biomarkers^12,13^. The visual assessment of TB by expert radiologists requires long training, is subjective, prone to errors and is subject to wide intra- and inter-expert variability. More importantly, it is extremely time-consuming, thus making it inappropriate for large studies^14^. Consequently, there is a need for quantification tools that are able to automatically, accurately and consistently compute CT imaging biomarkers. The initial step for their computation is to extract the lung from the chest CT volume^15^. This process is crucial, as a rough segmentation produces incorrect data that may reduce the accuracy of the disease burden quantification.

**Figure 1:**
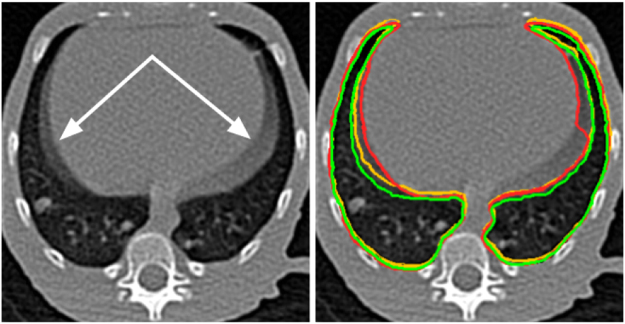
Sample slice from a chest CT volume of a subject infected with *Mycobacterium tuberculosis*. The presence of fuzzy boundaries (white arrow) caused by respiratory movement artifacts makes it difficult to delimit the lung boundary; (Right) The annotations performed by the experts are combined to explicitly illustrate the differences and shown with a red, yellow and green outline, respectively.

Segmentation of TB-infected lungs is especially complex in preclinical studies. The expected variability of the pulmonary inflation caused by the respiratory cycle is increased and less predictable than that of healthy subjects owing to the changes in lung compliance caused by the disease and to the breathing difficulties experienced by anesthetized infected animals. Moreover, CT image acquisition in TB animal models is usually performed on free-breathing animals to avoid the additional level of complexity added by the intubation in the manipulation of the animal, resulting in the presence of significant respiratory motion artifacts. This effect produces fuzzy boundaries, especially in the diaphragm area (Fig. 1), thus implying an uncertain delimitation of the lungs beyond the segmentation technique used.

Manual segmentation of the lungs is subject to especially wide intra- and inter-expert variability in the presence of those fuzzy boundaries^14^. Most of the state-of-the-art methods for automatic lung segmentation are not designed to deal with the specific problems present in Mtb-infected lungs under the presence of strong respiratory motion artifacts^15^. They generally are not able to differentiate between the neighboring soft tissue and the lesions attached to the pleura since their density (Hounsfield Units) is similar^16^. Well-known thresholding methods^17^ perform appropriately when extracting healthy tissue but cannot cope with HU variability. Region-based methods^18,19^ fail in the presence of abnormalities and are highly user-dependent, and atlas-based methods^20^ fail to obtain a suitable general model able to capture the singularity of the disease. The more recent approaches, which are mostly based on supervised learning methods^21^, require a large dataset labeled by an expert to ensure appropriate training and are not free from bias.

In this work, we present an automatic pipeline able to segment lungs infected with Mtb and place considerable importance on the robust and consistent identification of fuzzy boundaries.

## Materials

### Experimental Animals

Male cynomolgus macaques, aged 3 to 4 years, were obtained from an established UK breeding colony for these studies. Genetic analysis of this colony has previously confirmed the cynomolgus macaques to be of Indonesian genotype^22^. Absence of previous exposure to mycobacterial antigens was confirmed. All animal procedures and study designs were approved by the Public Health England Animal Welfare and Ethical Review Body, Porton Down, UK, and authorized under an appropriate UK Home Office project license. All animal procedures were performed on a facility with biosafety level 3 laboratories.

### Aerosol Exposure

Macaques were challenged by exposure to aerosols of Mtb as previously described^23,24^. Mono-dispersed bacteria in particles were generated using a 3-jet Collison nebuliser (BGI) and, in conjunction with a modified Henderson apparatus, delivered to the nares of each sedated primate via a modified veterinary anaesthetic mask. Challenge was performed on sedated animals placed within a ‘head-out’, plethysmography chamber (Buxco, Wilmington, North Carolina, USA) to enable the aerosol to be delivered simultaneously with the measurement of respired volume. The calculations to derive the presented dose (PD) (the number of organisms that the animals inhale) and the retained dose (the number of organisms assumed to be retained in the lung) have been described previously^23–25^.

### CT Imaging

Our dataset comprises 63 CT scans of the chest acquired from 9 different subjects at 7 time points (0, 3, 12, 16, 20, 24 and 28 weeks after aerosol exposure to Mtb). The subjects were treated with different combinations of antibiotics^24^. The chest CT scans were acquired with a 16-slice Lightspeed CT scanner (General Electric Healthcare, Milwaukee, WI, USA) with voxel spacing of 0.23 mm × 0.23 mm × 0.625 mm and in-plane resolution of 512 pixels × 512 pixels.

## Methods

### Automatic Lung Segmentation

**Figure 2:**
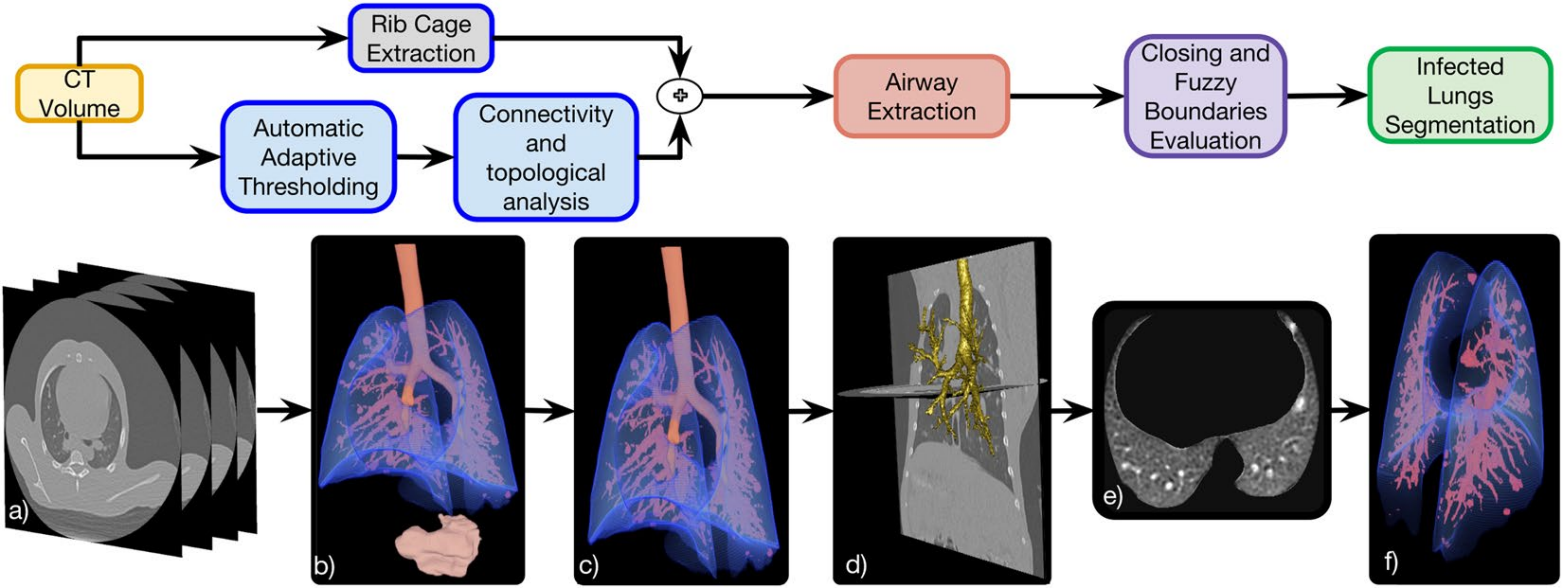
Automatic lung segmentation pipeline: (**a**) Source chest CT volume; (**b**) 3D rendering of the air-like structures detected in the image using automatic adaptive thresholding; (**c**) 3D rendering of the preliminary lung and connected airways segmentation obtained using a set of topological operations based on the position of all pre-segmented structures; (**d**) Isolated airways tree extracted with a propagating wavefront approach; (**e**) axial slice of the final lung segmentation in which the lesions caused by Mtb and attached to the pleura have been included and the motion artifacts discarded; (**f**) 3D rendering of the final lung segmentation including healthy parenchyma, the damaged parenchyma and the blood vessels.

The automatic lung segmentation pipeline is composed of three main steps, as depicted in Fig. 2 and explained in the following sections.

#### Preliminary Lung and Airway Tree Segmentation

*Automatic Adaptive Thresholding*: The first step goal is to obtain a rough segmentation of the lungs, including the airway tree, based on the well-known algorithm introduced by *Hu et al*.^17^. It separates air-filled structures (i.e., healthy parenchyma, stomach, airways, image background) from more dense tissues in the whole image volume (Fig. 2(a,b)). The two classes (air-like and non–air-like voxels) are identified by Otsu thresholding^26^ on the bimodal distribution of a chest CT volume histogram. *Rib Cage Extraction*: Although the literature contains robust approaches to rib cage and sternum segmentation^27–29^, it was not necessary for our purpose— and beyond the scope of the present study— to implement a highly accurate and time consuming segmentation. Instead, we use a simple technique, which although unable to capture the specific shape of each bone was good enough to establish a convex hull for the ribcage. First, we defined voxels with a value similar to the rib cage bones (over 900 Hounsfield units (HU)) as seeds. Then, we perform region-growing segmentation using the criteria given by the confidence connected segmentation method^30^.

*Connectivity and Topological Analysis*: In order to isolate the lungs from the rest of the segmented air-filled structures, as described in^31,32^, we utilized the differences in size and anatomical location of the secluded objects as follows: (a) excluding the objects located outside the convex hull formed by the partial extracted ribcage (Fig. 2(c)) and (b) selecting as lung tissue, the structures at the minimal Euclidean distance to the ribcage centroid (Fig. 2(d)).

#### Airway Tree Extraction

Due to the intricate morphology of the airway tree, a specific algorithm was needed to extract it from the overall lung volume (Fig. 2(d)). Our approach adapted a method based on modeling a propagating wavefront through the trachea, as introduced by *Schlathoelter et al*.^33^ and extended by *Bulöw et al*.^34^. In particular, we use the implementation described by *Artaecheverria et al*. and *Ceresa et al*.^35^, which introduced improvements in leakage detection. The complete airway tree extraction procedure is concisely explained in the Supplementary Material.

#### Morphological Closing and Fuzzy Boundaries Evaluation

The last step of the automatic lung segmentation procedure is a refinement process to include missing lesions attached to the pleura and to remove the fuzzy boundaries produced by the respiratory motion artifact.

**Figure 3:**
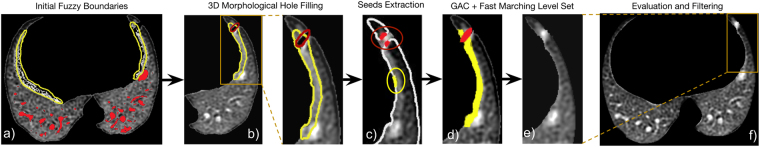
Lung segmentation evaluation workflow illustrated using a sample sagittal CT slice multiplied by its lung mask: (**a**) Axial slice of the segmented lung obtained after the Lung and Airway Segmentation and Airway Extraction processes showing holes (black areas inside the parenchyma) and fuzzy boundaries (in yellow); (**b**) Segmentation after the 3D morphological hole filling process including the holes enclosed by the lung parenchyma; (**c**) Seeds extracted on the eroded lung surface both in fuzzy boundaries (in yellow) and in TB lesions attached to the pleura (in red); (**d**) Respiratory motion artifact in the diaphragm area (in yellow) and TB lesion mask (in red) extracted by the combined level set and active contour approach; (**e**) Final segmentation in which the lesion attached to the pleura has been included and the fuzzy boundaries excluded.

*Morphological 3D Hole Filling*: Holes, defined as black voxels of the mask that are not connected to the boundaries of the lung segmentation, are removed with an iterative hole-filling filter using the approach described in *Janaszewski et al*.^36^ (see Fig. 3(b)). At each iteration, a neighborhood of the hole of (1 mm × 1 mm × 1 mm) was evaluated in order to add new voxels to the mask. It is important to remark that the parameters driving the morphological operations are fixed based on the prior knowledge about the subjects anatomy (see Experimental Animals) and its value is kept the same for all CT volumes.

*Fuzzy Lung Border Segmentation and Evaluation*: We specifically propose excluding movement artifacts and including lesions attached to the pleura in our lung segmentation using level sets and geodesic active contours^37^, which have proven successful in similar tasks^38,39^. First, the lung surface was extracted from the mask obtained after the morphological 3D hole-filling process: the lung surface was computed as the subtraction of the mask and an eroded version computed using a kernel of 1 *mm* radius. Then, to obtain automatically the seeds for the level-sets algorithm, we assumed that the fuzzy regions (lesions or respiratory movement artifacts) had the highest values at the lung boundary (see Fig. 3(b)). Therefore, the seeds are chosen to be the outliers of the intensity distribution at the previously delimited lung boundary (see Fig. 3(c)). We set a voxel, *v*_*i*_, as the seed based on the following criteria:

1 $${v}_{i}\in seeds\iff I({v}_{i})\ge {\mu }_{sp}+2.5{\sigma }_{sp}\,\forall {v}_{i}\in s{p}_{border}$$

where $I(\,\cdot \,)$ is the voxel intensity, *sp* represents the segmented lung parenchyma obtained as the output from the morphological hole-filling routine, *sp*_*border*_ corresponds to the boundary voxels, and *μ*_*sp*_ and *σ*_*sp*_ are the mean and standard deviation of the intensities of the voxels within *sp*_*border*_, respectively. Assuming a Gaussian distribution of the intensities and by setting beta to 2.5, we retain 1.3% of the voxels, in order to capture just a few reliable outliers.

These seeds were used to create the initial contours for the fast marching level sets. Note that several seeds could be placed at a given fuzzy boundary, but the level sets will expand evolving into complex shapes and merge since the intensity gradient is smooth. However, the level sets placed on the fuzzy boundaries do not merge with those placed on the lesion areas as can be observed in Fig. 3(c), where the intensity gradient was too large.

Several coarse level sets were obtained as output. These were used as initial contours (*x*_0_) for the geodesic active contour algorithm^37^. Namely, a contour was fitted to the region ruled by the following partial differential equation (PDE):

2 $$\frac{\partial {\rm{\Psi }}}{\partial t}=-\,\alpha {\bf{A}}({\bf{x}})\cdot \nabla {\rm{\Psi }}-\beta \,P({\bf{x}})|\nabla {\rm{\Psi }}|+\gamma Z({\bf{x}})\kappa |\nabla {\rm{\Psi }}|,$$

where Ψ is the level set, **x** is a point of the contour, **A**(**x**) controls the advection, *P*(**x**) is the propagation and *Z*(**x**) is the spatial modification of the mean curvature *κ*; *α*, *β* and *γ* are scalars which module each term of the contour evolution. Their value was heuristically set to *α* = 1.0, *β* = 0.25, *γ* = 2.0. The outputs were refined level-set contours for both the lesions and the fuzzy boundaries. Once the contours were determined, lesions were discriminated from artifacts based on the prior morphological information: contours with a sphericity over 0.85 were selected as lesions and included within the segmented lung (see Fig. 3(d)).

### Lung Segmentation Evaluation

The quality of the automatic segmentations for medical imaging applications is commonly estimated with respect to a manually or semi-automatically generated ground truth. The most commonly used evaluation measures are computed as an average of the intersected volumes between both segmentations (i.e., Dice similarity coefficient)^40,41^. For our application, good values of the measures could be misleading, if relatively small volumes at the fuzzy boundaries (i.e., lesions, respiratory motion artifacts) are incorrectly segmented. In those cases, the perceived decrease in quality as given by the measure will be minor but these errors in lung segmentation would generate considerable bias in subsequent quantification of disease burden.

To mitigate this issue in the evaluation of the goodness of the proposed lung segmentation method, we use the procedure described below to select the slices that most probably have fuzzy boundaries. Rough segmentations of the lungs were semi-automatically computed in 63 subjects using an in-house platform^42^ specifically created for the interactive segmentation of TB-infected lung. To segment the lungs using the platform, the user specifies at least 1 seed in the center of the left lung and right lung. The segmentation then propagates by means of a region-growing algorithm. The user can manually specify frontier surfaces to prevent the segmentation from reaching adjacent air-filled regions. The platform has added functionalities to enable manual correction of the results. Once the lungs are interactively segmented, the Hausdorff distances between the automatic lung segmentation obtained before and after the refinement step with respect to the semi-automatic segmentations are computed. The differences in the Hausdorff distances are due to the corrections performed by the refinement routine and pointed out to those slices in which the segmentation is more uncertain owing both to the variability introduced by each subject and to the disease course. We then choose the 156 slices with the largest differences in the Hausdorff distance to build a surrogate ground truth, as described in detail in the Supplementary Material.

Three experts interactively segmented the selected slices, paying particular attention to the boundary delimitation. The very accurate segmentations obtained were then combined by consensus to provide a surrogate ground truth. Characterization of the agreement, computing the intra-class correlation coefficient (ICC), between the lung segmentations performed by the experts showed excellent consistency (details can be found in Supplementary Material).

The individual expert segmentations and the surrogate ground truth are compared with the proposed method (referred to as refined -Ref-) and two other approaches intended for healthy or slightly damaged lung segmentation: the aforementioned manual segmentation (referred to as semi-auto -Semi-) and the traditional fuzzy connectedness–based lung segmentation (referred to as FC), which has a publicly available open-source software lung segmentation tool (http://www.nitrc.org/projects/nihlungseg/)^43^. For the latter, we used the best performing manual seeding mode, as recommended by the authors for refining segmented region maps, namely, filling holes with a 0.44 mm-diameter binary filter and checking fuzzy connectedness.

The similarity is measured as both volume overlap and distance between surfaces with the following metrics: Dice similarity coefficient (*DSC*), Hausdorff distance (*HD*), Hausdorff distance averaged (*HDA*), false-positive error (*FPE*), false-negative error (*FNE*) and volume dissimilarity (*VD*). The *HD* and *HDA* measures are indicators of a given method’s ability to delineate the tissue boundaries. The *FPE*, *FNE* and *VD* indexes provide additional information for the volume overlap measured by the *DSC*. In particular, *FPE* is related to over-segmentation, *FNE* to under-segmentation and *VD*, evidently, to volume differences.

In order to better understand the dispersion of the measures for this comparison, box plot charts for each similarity index are also obtained. The characterization of the dispersion of the similarity indexes is particularly interesting in our case owing to the complexity of the dataset used. We refer to each comparison between a method and the surrogate ground truth for a given similarity index specifying the method as sub-index (e.g., *DSC*_*Ref*_. refers to the median DSC of the comparison between the refined segmentation and the surrogate ground truth).

Finally, we studied the statistical significance of our results to assure the objectivity of our conclusions. For each evaluation metric and for each reference segmentation, the outputs of the three segmentation methods were compared using a paired t-test. A *p* value below 0.05 was considered statistically significant.

### Data availability

The dataset analysed during the current study are available from the corresponding author on reasonable request.

### Ethical approval

All animal procedures and study designs were approved by the Public Health England Animal Welfare and Ethical Review Body, Porton Down, UK, and authorized under an appropriate UK Home Office project license.

## Results

### Qualitative Results

**Figure 4:**
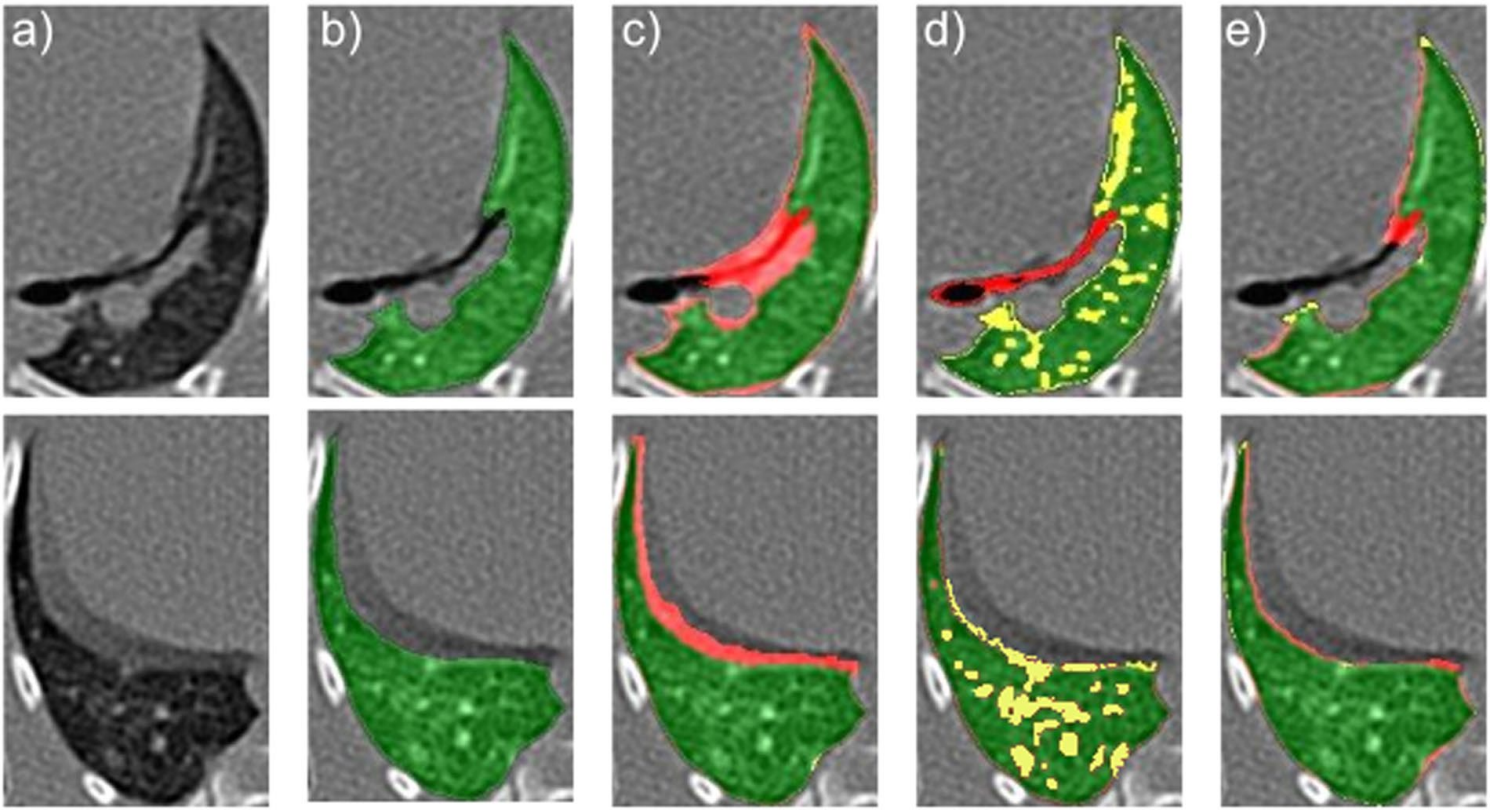
Sample lung segmentations on a representative slice (**a**) corresponding with the surrogate ground truth (**b**), the semi-automatic segmentation (**c**), the fuzzy connectedness segmentation (**d**), and our proposed method (**e**). The regions in which there is overlap with the surrogate ground truth are colored in green, the false-positive errors in red and the false-negative errors in yellow.

Figure 4 illustrates the computed lung segmentations on a representative slice from those retained (i.e., those in which the segmentation is most uncertain). The segmentations corresponding to the semi-automatic approach (panel c) are subject to over-segmentation: the delimitation of the lungs goes beyond the lung parenchyma including respiratory movement artifacts. As per the FC approach (panel d), we observed that a number of lesions, independently of their localization, were not included in the segmentation owing to the lack of sensitivity of the method to those areas. The amount of over- and under-segmentation (highlighted in red and yellow, respectively) caused by the proposed method was clearly reduced with respect to the other two approaches.

### Quantitative Results

**Figure 5:**
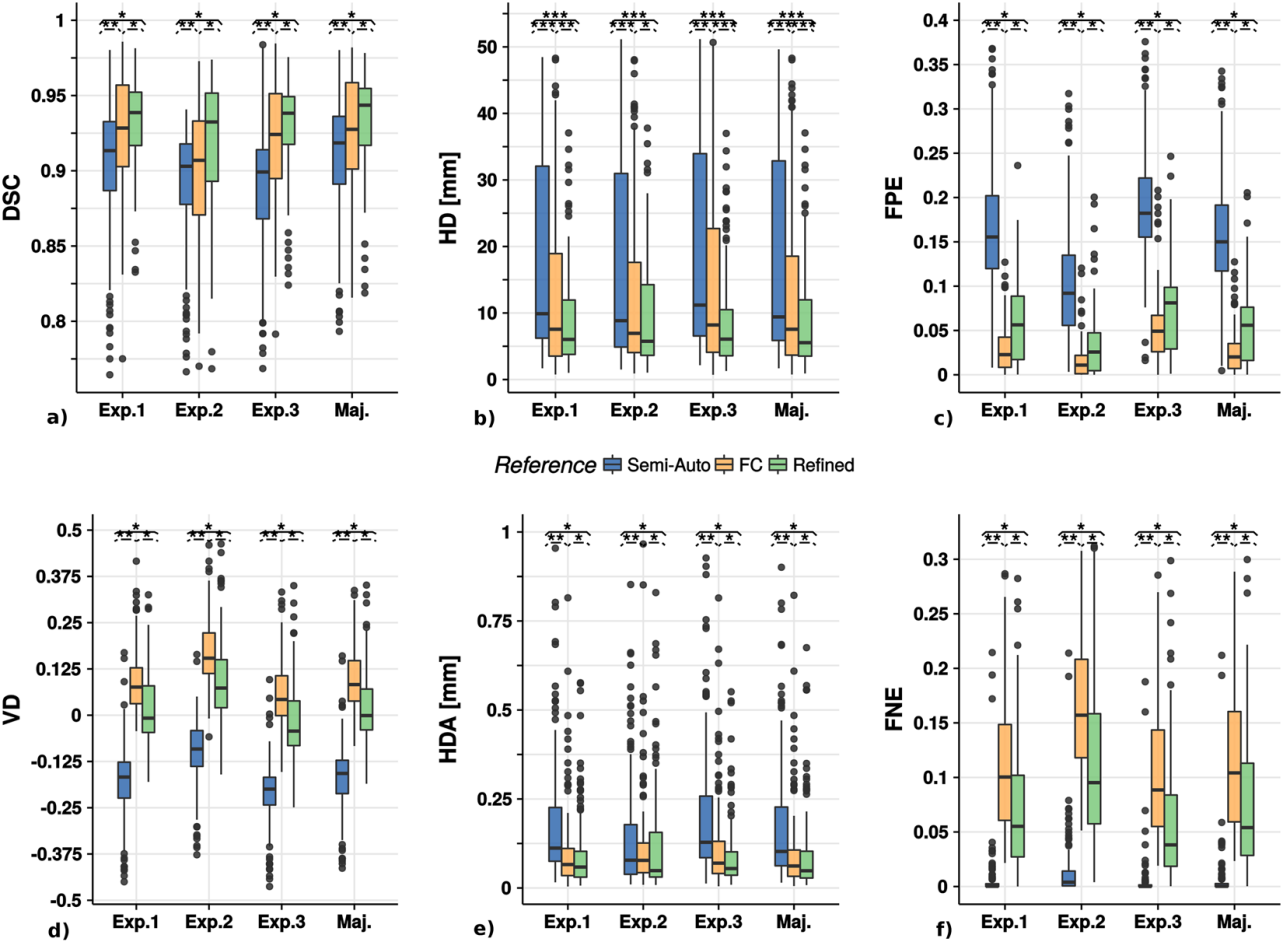
Boxplot charts for the similarity indexes: (**a**) Dice Similarity Coefficient (*DSC*); (**b**) Hausdorff Distance (*HD*); (**c**) False Positive Error (*FPE*); (**d**) Volume Dissimilarity (VD); (**e**) Hausdorff Distance Averaged (*HDA*); (**f**) False Negative Error (*FNE*). The lung segmentation obtained with the proposed method (refined) is compared with the semi-automatic (semi-auto) and the fuzzy connectedness approaches in the individual expert annotations (Exp. 1, Exp. 2 and Exp. 3) and the surrogate ground truth obtained by the expert consensus as explained in the Supplementary Material (Maj.). The asterisks over each group of boxes indicate statistically significant differences between the lung segmentation methods compared: $p < 0.05\equiv \ast$, $p < 0.01\equiv \ast \ast$ and $p < 0.001\equiv \ast \ast \ast$.

Figure 5 shows the box plot charts for each similarity index of the refined (Ref), the semi-automatic (Semi) and the fuzzy connectedness lung segmentation (FC) against the manual annotations performed by each expert (Exp. #) and the consensus surrogate ground truth (Maj.). The numerical results are provided in Table 3 of the Supplementary Material. The refined segmentation provides the most similar results with respect to the experts’ delimitation and, thus, with respect to the surrogate ground truth. In this sense, our method achieves the largest volume overlap, as reflected by the *DSC* (mean *DSC*_*Ref*_ = 0.933; median *DSC*_*Ref*_ = 0.943). The second best-performing method, the FC, which was intended for the segmentation of slightly infected lungs, presents a close mean *DSC* (mean *DSC*_*FC*_ = 0.926) but more distant median *DSC* (median *DSC*_*FC*_ = 0.922). Our method achieves much lower distances (*HD* and *HDA*) with respect to the surfaces of the surrogate ground truth than the others (between 1.2 and 5.1 mm with respect to the median (*HD*_*Ref*_ = 5.537 mm) and between 2.8 and 11 mm with respect to the average value (*HD*_*Ref*_ = 8.642 mm)). Our method presents similar rates of under- and over-segmentation, around 6%. In contrast, the semi-auto approach achieve a larger over-segmentation rate (median *FPE*_*Semi*_ = 15%, mean *FPE*_*Semi*_ = 16%) but a much smaller under-segmentation rate (median *FNE*_*Semi*_ = 0.2%, mean = 0.6%) while the FC method provide the opposite results (mean *FPE*_*FC*_ = 2.4%, median *FPE*_*FC*_ = 2.2%, mean *FNE*_*FC*_ = 11% and median *FPE*_*FC*_ = 10.4%). These imbalances make the differences between the volumes obtained by the experts (consensus) and those obtained with the semi-automatic and the fuzzy connectedness methods much higher than those measured for our approach. The volume dissimilarity index for the latter is close to zero in all cases (mean *VD*_*Ref*_ = 0.026, median *VD*_*Ref*_ = −0.0009). All the differences as illustrated in Fig. 5, are statistically significant except for the *HDA* index on the Refined and FC segmentations when Expert 2 is used as reference.

**Figure 6:**
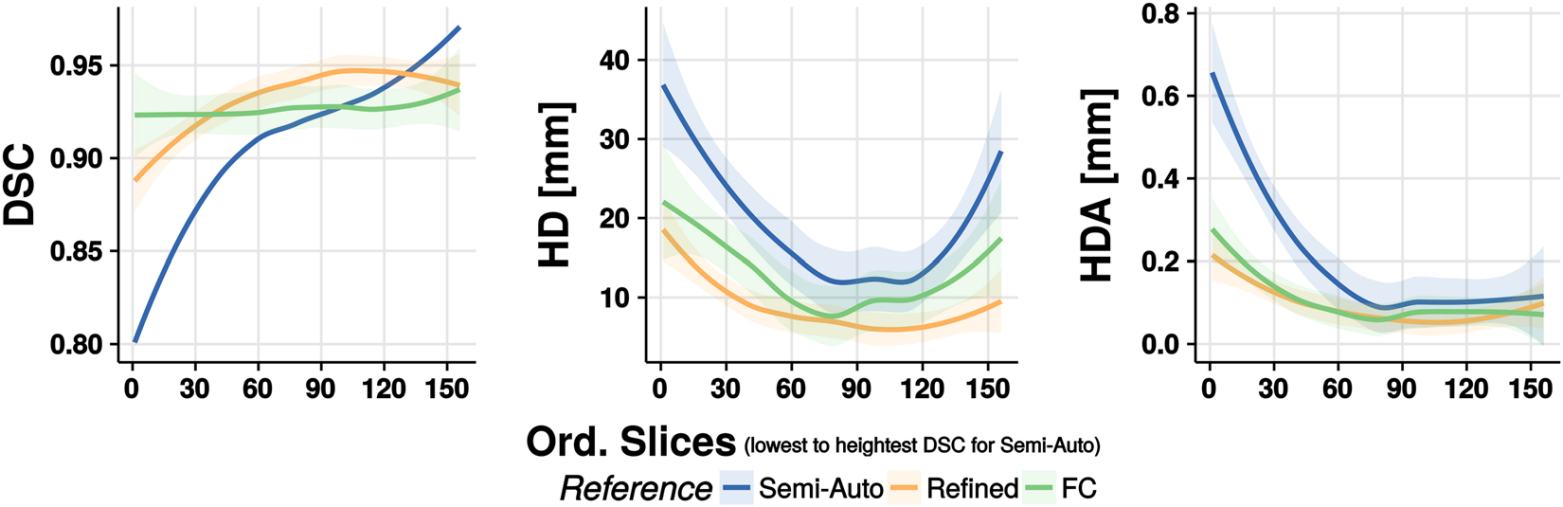
Dice similarity coefficient (DSC), Haussdorff distance (HD) and Haussdorff distance averaged (HDA) plots along the slices sorted in ascending order based on the DSC of the semi-automatic segmentation with respect to the surrogate ground truth. Data have been filtered with the locally weighted scatterplot smoothing (LOESS) model. The 95% confidence interval is drawn as a shadow of the same color as the corresponding line.

Figure 6 displays *DSC*, *HD* and *HDA* plots over the slices arranged in ascending order as given by the *DSC* of the semi- automatic segmentation with respect to the surrogate ground truth. The data have been filtered following the locally weighted scatterplot smoothing (LOESS)^44^ model in order to achieve a better appreciation of the patterns and the differences between the approaches. The *DSC* plot shows that the gap between the proposed and the semi-automatic method (about 10% for the first slice) decreases as we move towards higher *DSC* slice values, while the difference with the *FC* method remains more stable. The *HD* index corresponding to the proposed method is smaller than the one for the other methods for all the slices. The improvement is 5–10 mm with respect to the Semi-Automatic approach and 0.5–7.5 mm with respect to the FC approach. Finally, the *HDA* index exhibits an exponential decay for all the methods.

## Discussion

We present a novel method for the automatic unsupervised segmentation of Mtb-infected lungs on chest CT volumes. The experiments performed reveal important improvements when an input volume is processed through our pipeline. As could be expected from a method focused on improving boundary detection, the Hausdorff distance is significantly smaller than with other methods while, at the same time, it does present reasonably good results for the volume overlap measures. This behavior is explained by the ability to reject fuzzy boundary artifacts while retaining most of the damaged tissue (especially the lesions attached to the pleura). Since the Hausdorff distance computes the maximum among the minimal distances for the all points in the two surfaces compared, small changes when delimiting a complex shape (such as those generated by the diseased lung) result in large Hausdorff distance values. Fortunately, the boundaries created by our method are consistent and stable, and inaccuracies in the boundary delimitation are less frequent. Moreover, improved delimitation enables the target volume to be filled more accurately, as reflected in the DSC values.

In our context, where the lung segmentation is a preparatory step for the quantification of the TB lesions burden during the course of the disease, these small differences are vital. High-quality segmentation is particularly important in the early stages. The sensitivity given by the radiological images is especially important when assessing latent tuberculosis due to the small parenchymal damage associated with this stage of the disease. Therefore, the fact that the refined method achieves the lowest Hausdorff distance measured by far in almost all the slices (Fig. 6) is a major step towards the proper quantification of disease burden, even with the current dispersion of the measure. This dispersion is mostly due to the intrinsic noise inherent in the delineation of complex slices. Thus, it is likely to appear in any segmentation method including manual delineations^14^. In the Methodological Results Section in supplementary materials, the inter-agreement differences between the experts’ delimitation are presented and show a good intra-class correlation coefficient (ICC) for the overall surface delimitation (*HD* = 0.88, *HDA* = 0.85) and lower values for the volume indicators of performance (*DSC* = 0.74, *FPE* = 0.71 and *FNE* = 0.6). The fact that small variations in delineation produce large dissimilarity values is even more obvious for the Hausdorff distance averaged, although, as observed in Fig. 5, the values of this measure are much smaller than the plain Hausdorff distance. A considerable number of outliers are present owing to the constant relatively large distance that exists between the surfaces corresponding to pairs of compared segmentations at several of the slices within the data set.

The more conservative segmentations, ie., those provided by the fuzzy connectedness–based method and our proposal perform better in terms of *HD* and *HDA* (Fig. 4). Hence, the segmentations provided are more suitable for subsequent quantification of the TB lesion burden.

It is important to emphasize that our method achieves a good balance between-false positive and false-negative errors, in contrast to the semi-auto segmentation results, which show, on average, 15% over-segmentation. The lung segmentation includes fuzzy regions, which will contaminate the subsequent analysis. In contrast, the FC segmentation is excessively conservative. It presents a tiny percentage of over-segmentation and 15% false-negative errors on average for the most uncertain slices in the dataset, thus potentially generating a misleading evaluation of TB infection. Although the refined method balances out possible errors, it still exhibits 5% false-negative errors on average, which could still influence the quantification of disease burden although less severely than the FC method.

The information from the error types makes it possible to explain the volume dissimilarities shown in Fig. 5. The semi-auto method presents the previously mentioned problems of over-segmentation, which account for the almost parabolic shape of the HD when the DSC increases in Fig. 6. The method presents a limit (at the parabola vertex), from where the segmentation is unable to fill the region of interest without growing beyond, thus presenting a few slices with better overlap (DSC) than the proposed method at the expense of losing sensitivity at the boundaries. Consequently, the HD remains flat, between the 90th and the 120th slice, only to increase dramatically afterwards, while a suitable segmentation should decrease or, at least, keep a low constant distance. The HD plot for the FC method in Fig. 6 presents similar behavior to the semi-auto method albeit for different reasons. As illustrated with the examples in Fig. 4, the FC method misses an important part of the volume-of-interest, resulting in considerable volume dissimilarity (see Fig. 5). Although the DSC trend in Fig. 6 is flatter than the one corresponding to the semi-auto method, it also presents a parabola vertex, which indicates an inability to capture the intricate shape of the selected surrogate ground truth. In contrast, the refined method shows a negligible value of volume dissimilarity (see Fig. 5) and a much less marked parabola shape (see Fig. 6). To further improve the accuracy of the lung segmentation, it could be much more appropriate to use novel indicators of segmentation performance that are more closely associated with the ulterior quantification than the overlap and surface indicators, which are clearly of limited validity owing to the variability of human criteria during the segmentation process. To this aim, we have introduced a quantification method which makes use of the proposed pipeline for lung segmentation and that presents satisfactory results^45^.

Regarding the possible extensibility and use of the proposed framework on a particular animal model, the framework allows easy re-parametrization by fine tuning of the parameters (shown at Table 1, Supplementary Materials) to other models (e.g., mice, humans). In order to improve our results and extend the framework to the segmentation of extremely damaged lungs, recent literature points out to the use of artificial intelligence/deep learning techniques. These have shown promising results lately, but further developments are needed to cope with their common limitations like the loss of resolution^46^, which impede the proper identification of boundaries, and the need of a large refined ground truth^47^, which results quite difficult to obtain. Considering these recent techniques and working extensively on them, it would be possible to employ the segmentations obtained with the proposed framework to train larger datasets which nowadays are not available for our specific model

To conclude, we present a novel lung segmentation method that can address the particularities of TB-infected lungs, which, in a subsequent step, would be able to produce meaningful quantitative data on disease burden.

## Electronic supplementary material

Supplementary Material
