# Supplementary material for: Unsupervised CT Lung Image Segmentation of a Mycobacterium Tuberculosis Infection Model
Source: Sci Rep. 2018 Jun 28;8:9802. doi: 10.1038/s41598-018-28100-x (PMC6023884; doi:10.1038/s41598-018-28100-x)
Supplement: Supplementary file 1 — Supplementary Material [file 41598_2018_28100_MOESM1_ESM.pdf]

Supplementary Material

Unsupervised CT Lung Image Segmentation of a Mycobacterium Tuberculosis Infection Model

Pedro M. Gordaliza[1,2,+], Arrate Muñoz-Barrutia[1,2,+], Mónica Abella[1,2,3], Manuel Desco[1,2,3,4], Sally Sharpe[5], Juan José Vaquero[1,2,*]

[1]Universidad Carlos III de Madrid, Departamento de Bioingeniería e Ingeniería Aeroespacial, Leganés, Spain
[2]Instituto de Investigación Sanitaria Gregorio Marañón, Madrid, Spain
[3]Centro de Investigaciones Cardiovasculares Carlos III (CNIC), Madrid, Spain
[4]Centro de Investigación Biomédica en Red de Salud Mental (CIBERSAM), Madrid, Spain
[5]Public Health England, Microbiology Services Division, Porton Down, England
*juanjose.vaquero@uc3m.es
+These authors contributed equally to this work

Supplementary Material

Airway Tree Extraction

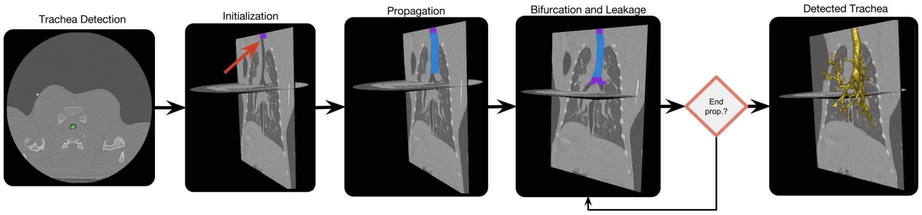

Figure 1: Airway tree extraction workflow. Step 1: Trachea seed detected by morphological analysis of the *HRCT* slices; Step 2: The trachea section is initialized by adding the neighboring voxels to the seed, thus creating a dome; Step 3: Spherical wavefront propagation ruled by the algorithm[1, 2]; Step 4: Check for bifurcations and leakages of the wavefront into the lungs; Step 5: The resultant isolated trachea after propagation.

Trachea detection and Initialization: The origin of the trachea is detected using a slice-by-slice search for the first isolated, air-filled area with a diameter from 5.5 to 8.5 mm (depending on the animal's weight [3]) and a roundness above 0.9. The center of mass (highlighted in green in Figure 1-Step 1) is chosen to be the seed to form a dome including the surrounding voxels and emulating a spherical wave.

Wavefront Propagation: The wavefront propagates, and the decision on whether to add voxels from the neighborhood is based on a 3D fast marching level set algorithm, which is ruled by the $time\ step$ and two thresholds as defined in *Artaecheverria et al.*[4, 5, 6]:

- $T_i = \mu_s + \alpha \cdot \max(\sigma_{s-1}, \sigma_{s-2})$ for the similarity between a voxel and its neighborhood, where μ_s is the mean intensity of the neighborhood voxel, σ_{s-1} y σ_{s-2} the standard deviation of two previous iterations and α the propagation factor.
- and T_s for the intensity gradient of the neighborhood computed using a Sobel filter.

After each propagation step, the dome shape is checked to detect possible bifurcations and the presence of leakages. If these are detected, the propagation of the current wavefront ends, thus defining a segment. If a bifurcation is found, two new wavefronts are initialized.

Bifurcation Detection: The algorithm exploits the expected wavefront shape to determine when a bifurcation occurs. The following rule is used: if $r_a > \beta \cdot r_e$, then mark that a bifurcation has occurred. r_a is the actual radius of the dome, r_e the expected radius and β a scalar factor (commonly chosen between one and two).

Leakage Detection: Owing to the presence of partial volume effects, beam hardening, motion artifacts or low-radiation induced noise, the contrast between the

airway lumen and the walls might become insufficient to guide the segmentation. Consequently, the wavefront could leak into the lungs. Two control mechanisms are implemented [6]: (a) The number of newly generated wavefronts after bifurcation is restricted to two, as a larger number generally indicates that several small segments are growing next to each other; this is a common indicator of leakage; (b) To be accepted, a fully grown segment needs to comply with three restrictions as measured by the growth rate, the compactness and the differences between wavefront sizes.

The *Growth Rate (GR)* indicator evaluates whether the waveform has propagated uniformly. It is defined as:

$$GR = \frac{1}{N}\sum_{i=1}^{N}\frac{|W_i|}{|W_{i-1}|} \quad < \quad T_{GR}, \tag{1}$$

where $|W_i|$ is the number of wavefront voxels at propagation step i, N the number of propagation steps and T_{GR} a threshold. Commonly, T_{GR} is chosen slightly larger than one.

The *Discrete Compactness* (c)[7] is computed as:

$$C = \frac{n - \frac{A}{6}}{n - (\sqrt[3]{n})^2} > T_C \tag{2}$$

where n is the number of voxels of the solid volume, A is the segment surface area and T_C a threshold defined to separate correct from incorrect segments. The typical range for T_C is $[0, 1]$.

Finally, the difference between the sizes of the last (W_{Last}) and the first (W_{First}) wavefronts is also computed and compared with a threshold T_W, because a large difference (over 10%) is a typical sign of leakage:

$$|W_{last} - W_{first}| < T_W \tag{3}$$

Surrogate Truth Extraction

Segmentation of lungs infected by Mycobacterium tuberculosis (Mtb) in chest computed tomography (CT) images is a complex task. Moreover, it is difficult to establish a suitable ground truth, as generation thereof is very time-consuming, subject to intra- and inter-expert variability, and prone to errors. As discussed, the commonly used measures of similarity do not represent well the unavoidable human variability inherent in a segmentation process. Therefore, in our workflow evaluation, we used a surrogate ground truth built as a consensus between three experts who performed detailed segmentations on 156 slices from our chest CT dataset. These slices were selected from the whole dataset using the procedure described in the next paragraph and were designed to ensure that the surrogate ground truth contains a representative sample of the most uncertain slices.

For the selection, we use the lung segmentation results obtained with the semi-automatic tool. This tool makes it possible to perform a simple interactive segmentation of each chest CT scan. Although the procedure is time-consuming and the results obtained are not ideal, they can be used as a reference to identify which of

the lung segmentations computed with our tool have changed more with the refinement procedure. To work with reliable segmentations, we exclude slices for which the DSC is below 0.7. In the subset, we measure the Hausdorff distance (pre-refinement and post-refinement), using the semi-automatic lung segmentation as a reference. Finally, we select the slices for which the absolute differences between the Hausdorff distances are larger than $\mu_{\Delta(HD_{pre}, HD_{post})} + 3\sigma_{\Delta(HD_{pre}, HD_{post})}$ (with μ and σ being the mean and standard deviation of the HD differences, Δ). In Figure 2, the HD differences are plotted against the DSC for all slices with a DSC larger than 0.7. The threshold is drawn in red, the slices with an HD difference under the threshold are shown in blue and those above in green. As observed, the $DSCs$ of the latter are uniformly distributed among all the possible DSC values, which is an indicator of disagreement at the surface delimitation and not at the complete filled volume.

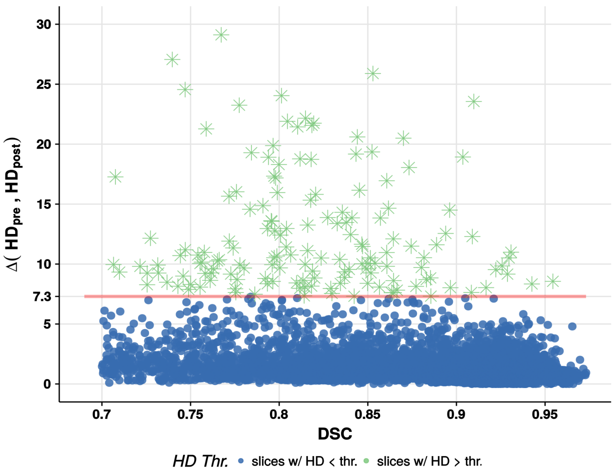

Figure 2: Hausdorff distance (HD) differences between those corresponding to the lung segmentation performed with our tool before and after the refinement process. HD was measured using the semi-auto segmentations as a reference. Only those slices with a Dice similarity coefficient (DSC) over 0.7 were included. HD corresponding to the slices for which the HD difference (Δ) is larger than a given threshold (thr., red line) are drawn in green, while points smaller than the threshold are shown in blue. In this case, the threshold is computed as $\mu_{\Delta(HD_{pre}, HD_{post})} + 3\sigma_{\Delta(HD_{pre}, HD_{post})}$ and the number of slices with an HD larger than the threshold is 156.

List of Used Parameter Values

Table 1 presents the list of used parameter values of the experiments described in the manuscript.

Section		Parameter	Value
Prel. Lung Segm.	Adaptive Thresholding	Otsu Threshold	Auto.
	Rib Cage Extraction	Seeds	> 900 HU
	Connectivity and Topological Analysis	Min. Object size	$10\ mm^3$
Airway Tree Segmentation	Trachea detection	Expected Perimeter	$5.5 - 8.5\ mm$
		Roundness	> 0.9
	Wavefront Propagation	$Time\ Step$	0.8
		T_i	-625 HU
		T_s	2.5
		α	1.4
	Bifurcation Detection	β	2
	Leakage Detection	T_{GR}	1
		T_C	0.72
		T_W	10%
Closing and Fuzzy Boundaries	Morphological 3D Hole Filling	$Kernel\ Radius$	$1\ mm$
	Fuzzy Lung Border Segmentation	α	1.0
		β	0.25
		γ	2
		sphericity	> 0.85

Table 1: Pipeline process (first column), algorithm (second column), parameters (third column) and their values (fourth column).

Inter-Expert Variability

In order to characterize the agreement between the lung segmentations performed by the experts, the intraclass correlation coefficient (ICC) of each similarity measure was computed. The semi-automatic segmentation was used as reference. In Table 2, the agreement coefficients are presented. We observed excellent consistency between the experts at the surface similarity measures (Hausdorff distance (HD), Hausdorff distance averaged (HAD), as was intended, and a good correlation for the volume overlap indicators (Dice similarity coefficient (DSC), false-positive error (FPE), false-negative error (FNE)). Figure 3 shows the boxplots corresponding to these results.

Coeff.	ICC	CI (95%)	p-val.
**HD**	0.88	0.84 to 0.90	<0.001
**HDA**	0.85	0.79 to 0.88	<0.001
**DSC**	0.74	0.66 to 0.81	<0.001
**FPE**	0.71	0.27 to 0.86	<0.001
**FNE**	0.60	0.26 to 0.77	<0.001

Table 2: Intra-class correlation coefficient (ICC) and 95% confidence intervals (CI) for the similarity coefficients between the three experts' delimitation and the refined masks. Note: Haussdorff distance (HD), Haussdorff distance averaged (HDA), Dice similarity coefficient (DSC), false-positive error (FPE), false-negative error (FNE).

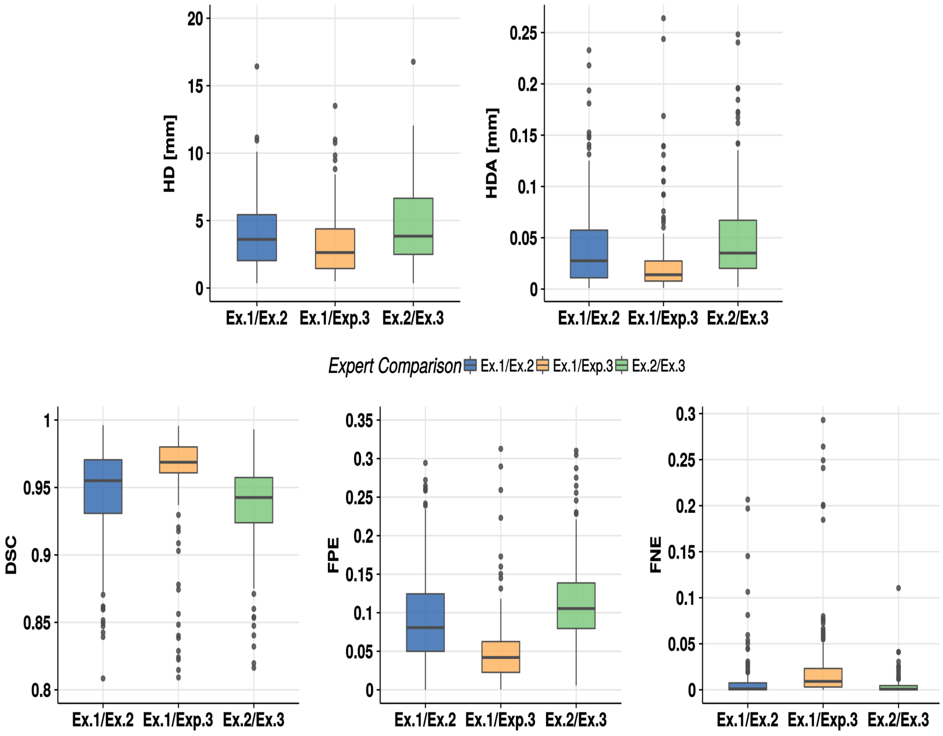

Figure 3: Boxplot charts for the similarity coefficients obtained from the 156 most uncertain slices in the experts' delimitations.

Overall Performance of the Automatic Lung Segmentation Methods

The overall performance of the refined, the semi-automatic and the fuzzy connectedness (FC) lung segmentation against the manual annotations performed by each expert (Exp. 1, Exp. 2 and Exp. 3) and the consensus surrogate ground truth (Maj.) are presented in Table 3.

Extra Qualitative Results

Figure 4 depicts the segmentation of the complete CT slices employed in the main text in order to present the qualitative results of the segmentation taking into account the fuzzy boundaries segmentation. Besides, the figure adds new examples which illustrates the performance of the proposed method.

Expert	Comparison	$\overline{DSC} \pm \sigma_{DSC}$	$\overline{HD} \pm \sigma_{HD}$	$\overline{HDA} \pm \sigma_{HDA}$	$\overline{FPE} \pm \sigma_{FPE}$	$\overline{FNE} \pm \sigma_{FNE}$	$\overline{VD} \pm \sigma_{Vol.Dis}$
Exp. 1	Semi-Auto	0.904 ± 0.04	18.741 ± 14.78	0.206 ± 0.25	0.167 ± 0.07	0.007 ± 0.03	-0.179 ± 0.10
	FC	0.926 ± 0.04	12.768 ± 12.79	0.105 ± 0.14	0.028 ± 0.03	0.112 ± 0.07	0.092 ± 0.08
	Refined	0.931 ± 0.03	8.801 ± 7.37	0.093 ± 0.11	0.059 ± 0.04	0.074 ± 0.06	0.017 ± 0.10
Exp. 2	Semi-Auto	0.891 ± 0.04	17.448 ± 14.66	0.159 ± 0.23	0.106 ± 0.07	0.013 ± 0.03	-0.101 ± 0.09
	FC	0.901 ± 0.04	12.346 ± 12.24	0.111 ± 0.13	0.015 ± 0.02	0.167 ± 0.07	0.170 ± 0.09
	Refined	0.920 ± 0.04	9.576 ± 7.95	0.117 ± 0.15	0.033 ± 0.04	0.118 ± 0.08	0.095 ± 0.11
Exp. 3	Semi-Auto	0.889 ± 0.04	19.835 ± 15.19	0.235 ± 0.27	0.193 ± 0.07	0.005 ± 0.02	-0.212 ± 0.09
	FC	0.919 ± 0.04	13.993 ± 13.44	0.116 ± 0.15	0.052 ± 0.04	0.105 ± 0.06	0.059 ± 0.09
	Refined	0.931 ± 0.03	8.825 ± 7.43	0.089 ± 0.09	0.059 ± 0.06	0.075 ± 0.05	-0.016 ± 0.10
Maj.	Semi-Auto	0.909 ± 0.04	18.674 ± 14.81	0.199 ± 0.25	0.16 ± 0.07	**0.006 ± 0.02**	-0.171 ± 0.09
	FC	0.926 ± 0.04	12.786 ± 12.85	0.103 ± 0.14	**0.024 ± 0.02**	0.116 ± 0.06	0.101 ± 0.08
	Refined	**0.933 ± 0.03**	**8.642 ± 7.36**	**0.091 ± 0.11**	0.054 ± 0.04	0.077 ± 0.06	**0.026 ± 0.09**

Table 3: Overall performance of the refined, the semi-automatic and the fuzzy connectedness (FC) lung segmentation against the manual annotations made by each expert (Exp. 1, Exp. 2 and Exp. 3) and the consensus surrogate ground truth (Maj.). Mean and standard deviation are provided for each index. For the surrogate ground truth, the best performing method is highlighted in bold for each index. Note: Dice similarity coefficient (DSC), Hausdorff distance (HD), Hausdorff distance averaged (HDA), false-positive error (FPE), false-negative error (FNE) and volume dissimilarity (VD).

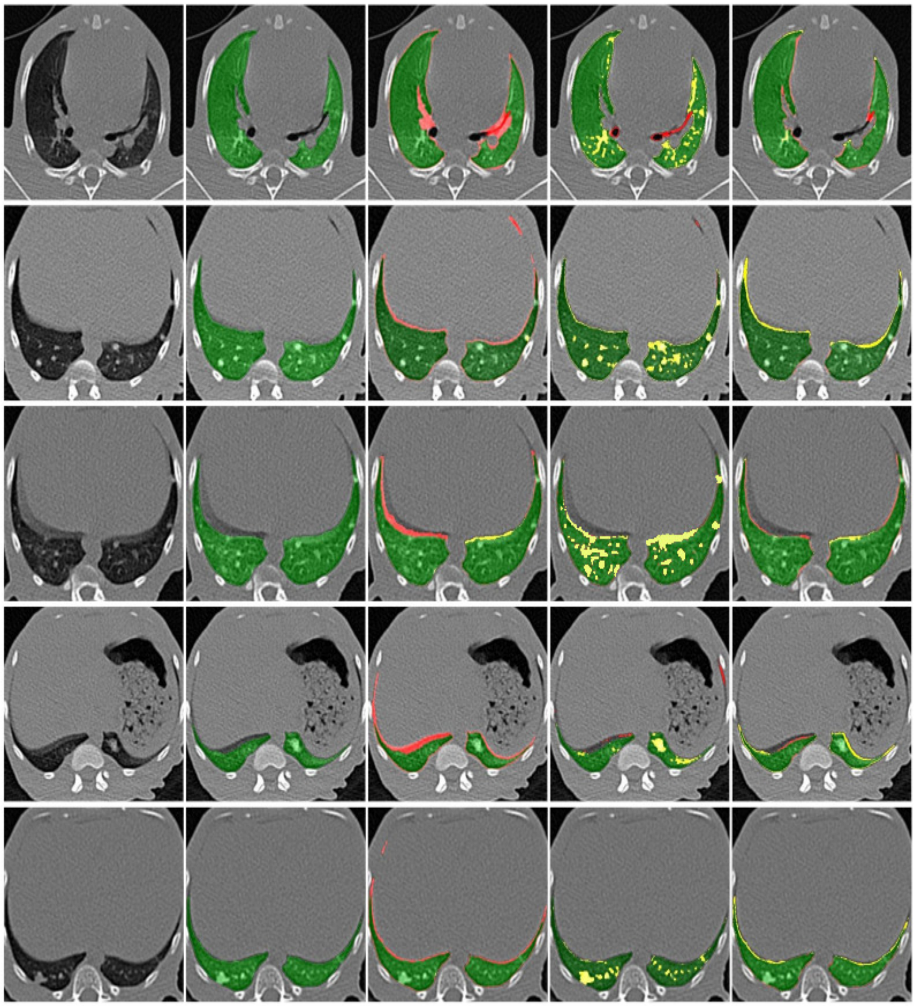

Figure 4: Sample lung segmentations on a representative slice (a) corresponding with the surrogate ground truth (b), the semi-automatic segmentation (c), the fuzzy connectedness segmentation (d), and our proposed method (e). The regions in which there is overlap with the surrogate ground truth are colored in green, the false-positive errors in red and the false-negative errors in yellow.

References

[1] T. Bülow, C. Lorenz, and S. Renisch, "A General Framework for Tree Segmentation and Reconstruction from Medical Volume Data," in *International Conference on Medical Image Computing and Computer-Assisted Intervention* (C. Barillot, D. R. Haynor, and P. Hellier, eds.), (Saint-Malo), pp. 533–540, Springer Berlin Heidelberg, 2004.

[2] T. Schlathoelter, C. Lorenz, I. C. Carlsen, S. Renisch, and T. Deschamps, "Simultaneous segmentation and tree reconstruction of the airways for virtual bronchoscopy," in *Proc. SPIE*, pp. 103–113, 2002.

[3] K. E. Pinkerton, L. S. Van Winkle, C. G. Plopper, S. Smiley-Jewell, E. C. Covarrubias, and J. T. McBride, "Architecture of the Tracheobronchial Tree," in *Comparative Biology of the Normal Lung*, pp. 33–51, Elsevier, 2015.

[4] T. Deschamps and L. D. Cohen, "Fast extraction of minimal paths in 3D images and applications to virtual endoscopy," *Medical Image Analysis*, vol. 5, no. 4, pp. 281–299, 2001.

[5] N. Forcadel, C. Le Guyader, and C. Gout, "Generalized fast marching method: applications to image segmentation," *Numerical Algorithms*, vol. 48, no. 1-3, pp. 189–211, 2008.

[6] X. Artaechevarria, D. Pérez-Martín, M. Ceresa, G. De Biurrun, D. Blanco, L. M. Montuenga, B. Van Ginneken, C. Ortiz-De-Solorzano, and A. M. Muñoz-Barrutia, "Airway segmentation and analysis for the study of mouse models of lung disease using micro-CT," *Phys. Med. Biol*, vol. 54, no. November, pp. 7009–7024, 2009.

[7] E. Bribiesca, "An easy measure of compactness for 2D and 3D shapes," *Pattern Recognition*, vol. 41, no. 2, pp. 543–554, 2008.
